# Determination of a Representative and 3D-Printable Root Canal Geometry for Endodontic Investigations and Pre-Clinical Endodontic Training—An Ex Vivo Study

**DOI:** 10.3390/dj11050133

**Published:** 2023-05-15

**Authors:** Michael Kucher, Martin Dannemann, Niels Modler, Robert Böhm, Christian Hannig, Marie-Theres Kühne

**Affiliations:** 1 Faculty of Engineering, Leipzig University of Applied Sciences, 04277 Leipzig, Germany; robert.boehm.1@htwk-leipzig.de; 2 Faculty of Automotive Engineering, Institute of Energy and Transport Engineering, Westsächsische Hochschule Zwickau, 08056 Zwickau, Germany; 3 Institute of Lightweight Engineering and Polymer Technology (ILK), Technische Universität Dresden, 01307 Dresden, Germany; niels.modler@tu-dresden.de; 4 Clinic of Operative and Pediatric Dentistry, Faculty of Medicine Carl Gustav Carus, Technische Universität Dresden, 01307 Dresden, Germany; christian.hannig@ukdd.de (C.H.); marie-theres.kuehne@ukdd.de (M.-T.K.)

**Keywords:** root canal morphology, endodontics, curvature, working width, length, tubular geometry, parametric model

## Abstract

Models of artificial root canals are used in several fields of endodontic investigations and pre-clinical endodontic training. They allow the physical testing of dental treatments, the operating of instruments used and the interaction between these instruments and the tissues. Currently, a large number of different artificial root canal models exist whose geometry is created either on the basis of selected natural root canal systems or to represent individual geometrical properties. Currently, only a few geometric properties such as the root canal curvature or the endodontic working width are taken into consideration when generating these models. To improve the representational capability of the artificial root canal models, the aim of the current study is therefore to generate an artificial root canal based on the statistical evaluation of selected natural root canals. Here, the approach introduced by Kucher for determining the geometry of a root canal model is used, which is based on the measurement and statistical evaluation of the root canal center line’s curvatures and their cross-sectional dimensions. Using the example of unbranched distal root canals of mandibular molars (n = 29), an artificial root canal model representing the mean length, curvature, torsion and cross-sectional dimensions of these teeth could be derived.

## 1. Introduction

In endodontics, there are several applications for artificial root canal models. In general, they are used for two main purposes: (1) pre-clinical endodontic training and (2) endodontic investigations. The endodontic training is designed for dental students to learn practical skills [1,2,3,4,5,6,7,8,9,10,11,12]. The aim is to develop the student’s own skills in realistic treatment situations as a preliminary step to treatment of selected patients in the student course. This preparation of the students for the practical work on the patients, such as the teaching of root canal instrumentation and obturation using transparent polymers [3], has been established for several decades.

In the field of endodontics, individual natural and artificial root canal models are used for the measurement and visualization of activated irrigation [13,14,15,16,17,18,19,20,21,22,23,24,25,26,27,28,29,30,31,32], for fatigue analysis of rotary endodontic files [33,34,35,36,37], for the quantification of dentin removal and canal transportation during a root canal preparation as well as irrigant activation including bacterial decontamination [28,38,39,40,41,42,43,44,45,46,47,48,49,50,51,52,53,54,55,56], for the analysis of the microbial behavior [57,58], for the investigation of obturation [59,60] or as input parameters for stress–strain analyses of teeth [61]. Hülsmann summarized different experimental models for studies on root canal preparation [46]. Gulabivala et al. reviewed root canal models that could be used to analyze fluid mechanics during root canal irrigation [18]. Different manufacturers and suppliers of dental equipment provide artificial root canal models, mostly made of transparent, colored or transparent and colored polymers for teaching and research purposes. Commercially available examples of these are artificial teeth [2,6,11,46], polymeric training blocks [18,31,47] and polymeric sub models of jaw areas [46]. Furthermore, there are examples of using simplified root canal models to investigate the behavior of human dental pulp spheres on dentin in vitro such as those carried out by Neunzehn et al. [62]. In endodontic research and for the realization of complex root canal geometries, additive manufacturing techniques are often used [5,6,7,50,63]. As an example, Kolling et al. [63] used 3D printing technology to create a novel root canal model with an actual fine anatomic root canal structure for student training. However, the students still favored extracted human teeth over 3D-printed teeth in terms of their physical properties when performing endodontic treatments in the simulation lab.

The particular geometry of these root canals is obtained from selected natural root canal systems [2,6,11,46] or to represent individual geometrical properties, such as different Schneider angles (compare, e.g., [48]). Currently, only a few geometric properties such as the root canal curvature [34,64] or the endodontic working width [65] are considered to generate these models. To describe the entire root canal geometry, Peters et al. [66] determined a volume model of maxillary molars by means of a rod-like structure, Dong et al. [67] used an elliptical cross-sectional shape to obtain 3D root canal models and Dannemann et al. [65,68] introduced an approach for a mathematical description by means of elementary parameters.

To improve the representational capability of the artificial root canal models, the aim of the current study is therefore to demonstrate an approach for the determination of a standardized artificial root canal and to generate an output file for 3D printing based on the statistical evaluation of selected natural root canals as introduced by Kucher [23]. Using 3D imaging data from [64,65], a virtual artificial root canal model for 3D printing based on average statistical values of distal root canals from a set of extracted human molars was obtained for the first time. This approach was based on 3D imaging data from a micro-computed tomography (µCT) scanning system. The obtained volume models of the selected unbranched root canals are used to measure and statistically evaluate the root canal’s length, curvature, torsion and cross-sectional dimensions. Using the mean values of these evaluations, the root canal center line and their cross-sections are reconstructed. Using the example of unbranched distal root canals of mandibular molars, an artificial root canal model representing the mean length, curvature, torsion and cross-sectional dimensions of these teeth is derived.

## 2. Materials and Methods

### 2.1. Artificial Root Canal Models and Purpose of Use in Endodontics

**Figure 1:**
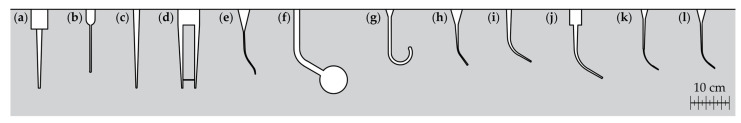
Selection of artificial root canal model’s geometries from different studies (obtained by reverse engineering approach): (**a**) Boutsioukis et al. [14], (**b**) Jiang et al. [20,21,22,70], (**c**) Macedo et al. [25], (**d**) Swimberghe et al. [29], (**e**) Kim et al. [56], (**f**) Gündoğar and Özyürek [35], (**g**) Kirsch et al. [34], (**h**) Pachpore et al. [71], (**i**) Roda-Casanova et al. [33], (**j**) Swimberghe et al. [30], (**k**) Silva et al. [55], (**l**) Huang et al. [47].

As mentioned above, there are several manufacturers, supplies and researcher groups which provide or use artificial root canal models. The artificial tooth models and training blocks for endodontic teaching purposes show different dimensions (see Figure 1) and are made of different materials (compare Table 1). Additionally, Dong et al. [67] and Dannemann et al. [68] have introduced approaches which are not included in Figure 1.

A very realistic approach for the preparation of natural teeth is demonstrated by Malentacca et al. [26], who prepared teeth with alcohol solutions to ensure that the dentine lost its opacity and the roots became transparent. As mentioned by Reymus et al. [6], tooth replicas should be able to simulate human dentine from the perspectives evaluated, i.e., properties such as radiopacity, micro-mechanical properties and hardness (compare, e.g., [69]). However, the focus of the current study is to demonstrate an approach for the determination of the geometry of an artificial root canal model.

**Table 1:** Artificial root canal models and purpose used in endodontics. The columns of the purpose of use indicate the nature of the work (x). The considered canal shapes are divided in curved ‘c’ and straight ‘s’.

Author(s), Ref.	Purpose of Use					Material	Canal Shape
	Endodontic Training	Dentin Removal	Activation Irrigants	Fatigue Instruments	Dental Tissue Regeneration		
Al-Sudani and Basudan [12]	x					Transparent or colored resin, such as acrylic resin	c
Bitter et al. [11]	x						c
Bürklein et al. [52]		x					c
Cassim and van der Vyver [51]		x					c
Christofzik et al. [50]		x					c
Gu et al. [54]		x					c
Hasselgren et al. [10]	x						c
Hasselgren and Tronstad [9]	x						c
Huang et al. [47]		x					c
Khalilak et al. [45]		x					c
Kim et al. [56]		x					c
Luz et al. [8]	x						c
Reymus et al. [7]	x						c
Reymus et al. [6]	x						c
Reymus et al. [5]	x						c
Shi et al. [42]		x					c
Silva et al. [55]		x					c
Sonntag et al. [41]		x					c
Spenst and Kahn [3]	x						s, c
Tchorz et al. [2]	x						c
Yekta-Michael et al. [1]	x						c
Alghamdi et al. [53]		x				Natural tooth	c
Al-Sudani and Basudan [12]	x						c
Bitter et al. [11]	x						c
Castagna et al. [15]			x				c
Eggmann et al. [17]			x				c
Gümüş and Delikan [19]			x				c
Hartmann et al. [49]		x					c
Hilaly Eid and Wanees Amin [48]		x					c
Loroño et al. [24]			x				c
Malentacca et al. [26]			x				c
Ni et al. [61]		x					c
Pawar et al. [60]			x				c
Peters et al. [44]		x					c
Retsas et al. [43]		x					c
Rodrigues et al. [28]		x	x				c
Yekta-Michael et al. [1]	x						c
Al-Obaida et al. [37]				x		Stainless steel	c
Boutsioukis et al. [14]			x				s
Chi et al. [36]				x			c
Gündoğar and Özyürek [35]				x			c
Kirsch et al. [34]				x			c
Conde et al. [16]			x			Manufactured dental hard tissue	s
Jiang et al. [70]			x				s
Jiang et al. [20]			x				s
Jiang et al. [21]			x				s
Jiang et al. [22]			x				s
Betancourt et al. [13]			x			Glass	s
Jiang et al. [22]			x				s
Swimberghe et al. [29]			x			Polymethylmethacrylate	s
Swimberghe et al. [30]			x				c
Macedo et al. [25]			x			Polydimethylsiloxane	s
Nagahashi et al. [27]			x			Porcine tooth	s
Neunzehn et al. [62]					x	Bovine tooth	s
Robberecht et al. [4]	x					Ceramic	s

### 2.2. Teeth Selection and Preparation

A number of twenty-nine extracted human mandibular first and second molars were collected from an oral and maxillofacial surgery clinic as well as from private dental practices. These teeth were included in the current study for determination of geometry of the artificial root canal model. The investigated teeth were extracted for medically justifiable reasons that were not connected to the current study. For these teeth, the patients’ sex, age, name, or general health condition were not taken into consideration. All teeth were cleaned from calculus, soft tissue and hard tissue. Only unprepared teeth with a completely intact distal root canal were used for these examinations.

### 2.3. Computed Tomographic Imaging Technique for the Determination of Root Canal Geometry

The tomographic imaging was performed by means of a µCT scanner (in situ CT FCTS 160 IS; Finetec GmbH, Garbsen, Germany) and remained ex vivo at all times. All measurements were performed using the following imaging parameters of the µCT system: tube voltage 80 kV, tube current 0.08 mA, exposure time 900 s, source object distance 150 mm and resolution 0.021 mm/pixel. The volume models of the individual root canals were determined by using a suitable grayscale threshold guarantying the determination of the root canal morphology and ensuring a low image noise. Firstly, image registration was carried out using the software for analyzing 3D measuring data (GOM Inspect 2018, Metiris, Gebenstorf, Switzerland). For the registration of the molars, the $x_{1}x_{2}$ plane was rotated parallel to the tooth occlusal surface and the $x_{2}x_{3}$ plane was aligned parallel to the tooth’s lingual view. The tooth height was denoted as $x_{3}$, the canal width as $x_{2}$ and the canal thickness as $x_{1}$.

### 2.4. Approach for Determining the Root Canal Model

**Figure 2:**
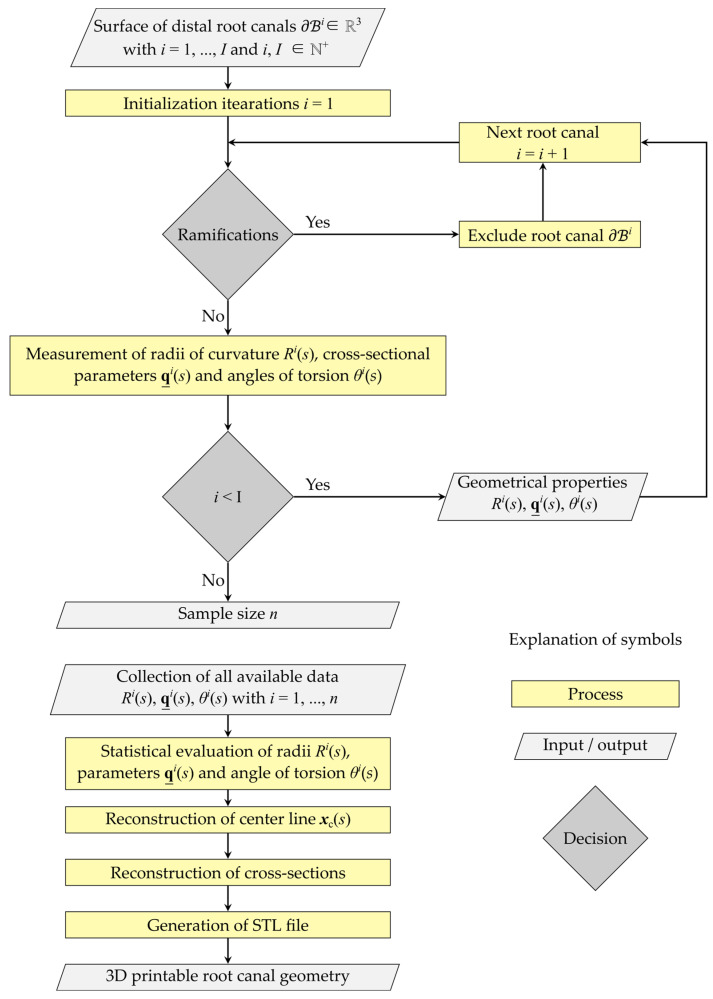
Flow chart of the approach for determining the 3D-printable artificial root canal model based on computer tomographic 3D imaging data.

The determination of the artificial root canal model is based on the calculation of the individual distal root canal’s radius of curvature, the measurement of the root canal cross-section, the statistical evaluation of these properties and the reconstruction of the geometrical-based artificial root canal model (Figure 2). The required sub-steps are described in the following.

### 2.5. Determination of Representative Center Line and Approximation of Cross-Sectional Dimensions

Distal root canals with ramifications were excluded from the evaluation procedure (Figure 2). The volume model was cut parallel to the root canal’s height axis defined as direction $x_{3}$ with a length between the individual segments of $∆x_{3}$ = 10 mm, which resulted in a number of $N_{j}$ slices. According to the description of Kucher et al. [64], the center of mass of each segment was calculated. The assembly of these points $x_{m}(s)$ gives the center line of the root canal with the canal length s and the radius of curvature

(1) $$R\left(s\right)=\frac{1}{\kappa \left(s\right)}=\frac{{\left|x_{m}^{\prime }\left(s\right)\right|}^{3}}{\left|x_{m}^{\prime }\left(s\right)\times x_{m}^{\prime \prime }\left(s\right)\right|}.$$

This procedure was repeated for all distal root canals without ramifications and leads to radii $R^{i}$(s).

**Figure 3:**
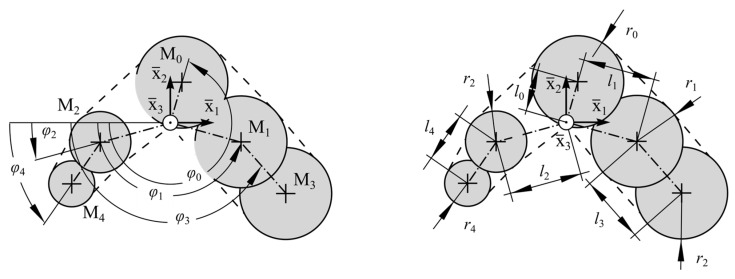
Parametric model for the approximation of the root canal’s cross-sectional dimensions based on Ref. [65].

Analogously, the root canal segments were used to measure the dimensions of the width and thickness of the individual cross-sections. Therefore, a parametric model as introduced by Kucher et al. [65,68] was used. This so-called Five Circle Model represents the convex hull of 5 circles (see Figure 3).

This model is described by means of 15 parameters ${\underline{q}}^{i}$, 5 angles $\varphi_{0}$, $\varphi_{1}$, $\varphi_{2}$, $\varphi_{3}$, $\varphi_{4}$, 5 radii $r_{0}$, $r_{1}$, $r_{2}$, $r_{3}$, $r_{4}$ and 5 distances $l_{0}$, $l_{1}$, $l_{2}$, $l_{3}$, $l_{4}$, respectively. Using an optimization algorithm, the parameters ${\underline{q}}^{i}$ were determined, from which we obtain the best approximation of the root canal cross-section (compare [65]). This approximation was carried out for all root canal segments. The used root canal model was able to approximate short oval, long oval, ribbon-shaped curved, irregularly bordered root canals (compare [23]). The local coordinate system ${\overset{-}{x}}_{i}$ of the parametric model was located at the related point of the center line $x_{m}(s_{j})$ at the location $s=s_{j}$. Thus, it follows that the parameters ${\underline{q}}^{i}$(s) depend on the canal length s.

### 2.6. Statistical Evaluation of Curvature and Cross-Sectional Measures

Because the radius of curvature is a critical value for the consideration of dentin removal [28,41,43,44,45,47,48,49,50,52,53,61] and endodontic instruments’ fatigue [34,35,36,37], the confidence interval (CI) computed at the 95% level was calculated for the radii of curvature $R^{i}$(s). This yields the lower CI of the curvature’s radius R(s). Using the arithmetic mean, the model parameters ${\underline{q}}^{i}$(s) of the individual distal root canals were averaged for the calculated number of root canals n without ramifications which were selected for the statistical evaluation. Due to the registration of each distal root canal (see Section 2.2), the model parameters ${\underline{q}}^{i}$(s) were normally distributed around their mean values. This averaging leads to the average model parameter $\underline{q}(s)$ which was used to reconstruct the geometry-based center line $x_{c}$ and cross-sections of the investigated distal root canals n. It should be noted that all root canals had different lengths $l^{i}$. Thus, the average length l was considered and each radius of curvature up to a length s≤l was used for the calculation of the mean.

### 2.7. Determination of the Root Canal’s Angle of Torsion

The included angle of torsion θ between the cartesian standard basis $e_{1}$ and the connecting vector $v_{34}$ of the two outer circle centers of the parametric model $M_{3}$, $M_{4}$ is used to describe the torsion of the root canal cross-sections in the $x_{1}x_{2}$ plane. Considering this angle, the root canals’ torsion can be calculated. Analogously to the root canal curvature and the model parameter, the average angle of torsion θ(s) was calculated using the individual root canal’s angle of torsion $\theta^{i}$(s).

### 2.8. Reconstruction of Geometry-Based Center Line and Cross-Sections

**Figure 4:**
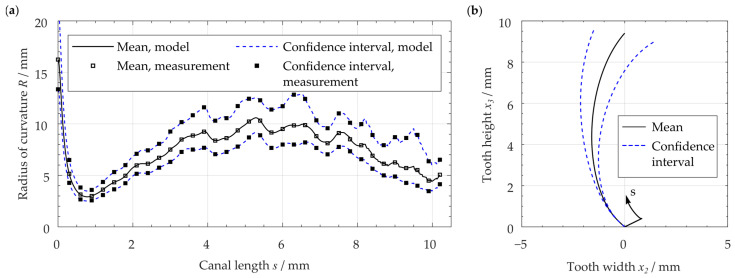
(**a**) Comparison of measured averaged radius of curvature and radius of the reconstructed center line, (**b**) reconstruction of average center line of the investigated distal root canals.

The reconstructed center line $x_{c}$ had the length equal to the average length l of all considered distal root canals. According to Kucher et al. [64], the center line of distal root canals is curved in both transverse coordinate directions $x_{1}$ and $x_{2}$. The proportions of these curvatures of the 3D space curve depend on the choice of the global coordinate system $x_{i}$. By determining the radius of curvature R(s) using Equation (1), a measure of curvature is obtained that is independent of the registration of the investigated root canal. The geometry-based canal center line was determined by connecting circular arcs each with an arc length of ∆s = 0.01 mm and the lower CI curvature’s radius of R(s) at location $s=s_{j}$. The center line was rotated with respect to the $x_{1}$ direction so that the canal’s start and end were at the position $x_{2}$ = 0 (cf. Figure 4).

The cross-sections of the artificial root canal model were obtained by average parameters $\underline{q}(s)$ at each location $s_{j}$. The local coordinate system of each reconstructed cross-section was determined, starting from the points of the reconstructed center line $x_{c}$ (cf. Figure 3). All cross-sections were rotated to represent the root canal’s torsion by the average angle of torsion θ(s). The center of the middle circle $M_{0}$ represents the center of the rotation (compare Figure 3). The combination of the geometry-based center line, the average torsion and the reconstructed cross-sections result in the artificial root canal model.

### 2.9. Generation of STL File for 3D Printing

The 3D data were imported into the 3D measuring software (GOM Inspect 2018, Metiris, Gebenstorf, Switzerland) as a point cloud. The point cloud was automatically polygonized. The resulting mesh was post processed and improved by using the built-in functions, such as smoothing and the automated corrugation of mesh errors. Then, the file was exported to the “Standard Triangle Language” (STL) file format which is native to the stereolithography computer-aided design (CAD) software created by conventional 3D systems. This data file can be used as a basis for the realization of replicates of the determined artificial root canal model.

**Figure A1:**
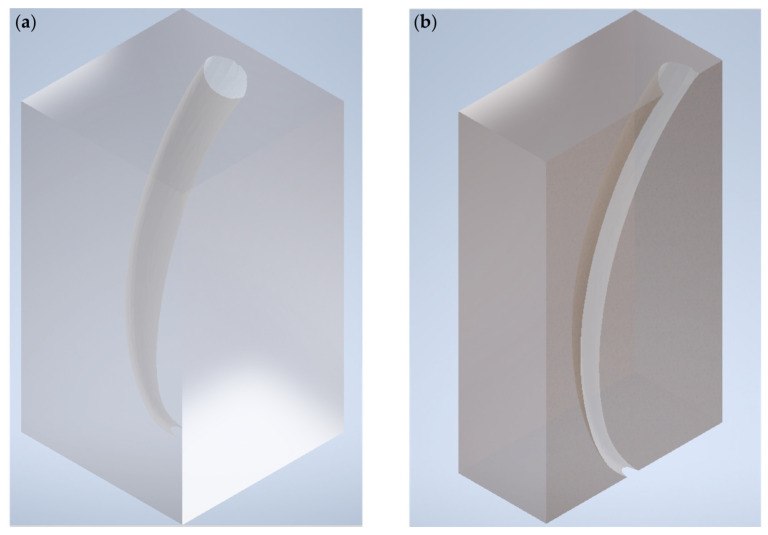
(**a**) CAD model of resulting artificial root canal model and (**b**) half cut of this model.

To create an endodontic training block, a volume model is first created from the STL file of the root canal model using a conventional CAD program. The root canal’s volume model is subtracted from a cuboid with the desired dimensions of the training block. The resulting solid represents a training block with a through-hole in the shape of the root canal (compare Figure A1). This model can be exported as an STL file to be printed with any conventional 3D printer.

## 3. Results

### 3.1. Resulting Reconstructed Center Line and Root Canal’s Angle of Torsion

The reconstructed center line of the examined distal root canals shows a c-shaped configuration (Figure 4). For the center line, the average length of 10.2 mm was considered. The center line has a minimum radius of curvature of R = 2.5 mm in the root canal’s apical region (compare Table 2). Using the curvature measurement method as used by Kucher et al. [64], a good agreement of the average radius of curvature and the reconstructed center line’s radius of curvature can be seen. Thus, it can be demonstrated that the radii are equal.

**Figure 5:**
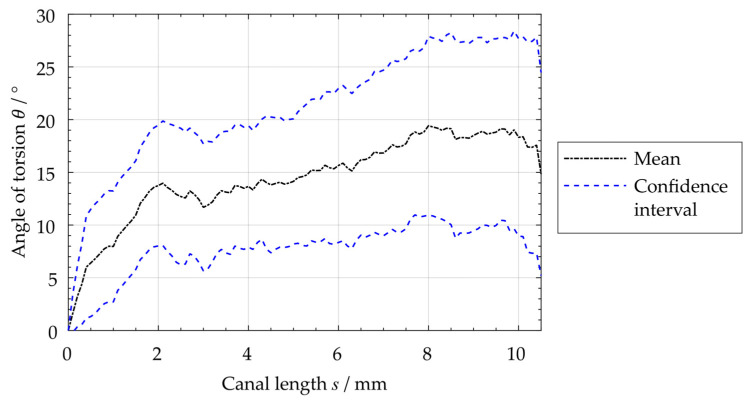
Measurement of root canal’s average angle of torsion.

Using the curve of the angle of torsion of the individual investigated distal root canals $\theta^{i}$(s), the upper CI of the torsion angle θ(s) was determined (Figure 5). This angle reaches a maximum value of 28.2° for the average torsion and decreases in the coronal region starting at a value of s > 10 mm.

### 3.2. Resulting Geometry-Based Artificial Root Canal Model

**Figure 6:**
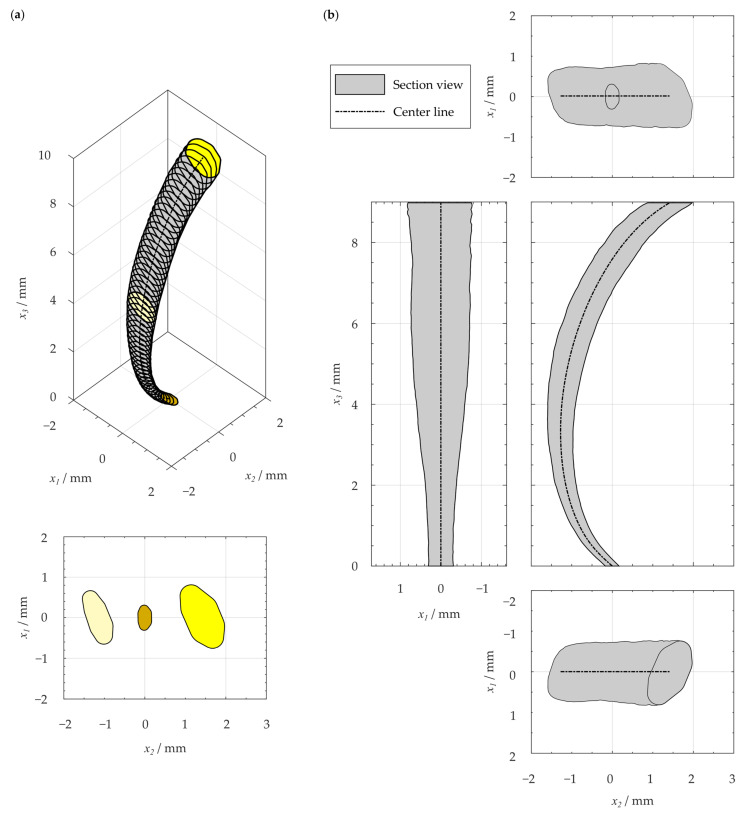
Geometry-based artificial root canal model based on statistical evaluation of distal root canals of human mandibular molars: (**a**) isometric view and projections of the base, medial and cover surface, (**b**) views according to projection method 1 [72]. Adapted with permission from Ref. [23]. Copyright 2023, Michael Kucher.

Taking the reconstructed center line into consideration, the resulting artificial root canal geometry represents a tubular structure with elliptical to irregularly ribbon-shaped, straight cross-sections, which are twisted starting from the base surface (Figure 6). The gradient of twisting is greatest in the apical region. The main dimensions in the transverse directions result in a ratio of the cross-section’s long axis to its short axis of 1.84 at s = 0 and a value of 1.94 at locations s=l. The mean taper of the canal’s short width has a value of 4.3% in the apical third 0≤s≤l/3 and 5.3% for the whole reconstructed root canal. The apical cross-section has an almost elliptical shape (compare Figure 6). In the resulting artificial root canal model, the length of the long axis of 0.62 mm and the length of the short axis of 0.36 mm were measured using an elliptical fit.

## 4. Discussion

The demonstrated approach for the determination of a geometry-based artificial root canal model was applied to the particular kind of distal root canal. For the capturing of the 3D root canal morphology, µCT scans of mandibular molars were used. As mentioned by Kucher et al. [64,65], the imaging process could also be carried out by means of cone beam computed tomography (CBCT). Considering these studies, it seems to be possible that the demonstrated approach is applicable based on the evaluation of CBCT scans. In this way, already existing imaging data from previous 3D investigations and in situ investigations can be realized. 

The particular geometry of root canal models is obtained from selected natural root canal systems [2,6,11,46] or represents individual geometrical properties, with different Schneider angles (compare, e.g., [47]), the radiologically determined two-dimension radius of curvature [34] or the endodontic working width [65] being considered to generate these models. Most of the investigated root canal models show an apical diameter between 0.15 and 0.8 mm, a taper between 2 and 6% and a length in the range from 16 to 20 mm [14,20,21,22,25,29,30,33,34,35,47,55,56,70,71]. The geometry-based root canal model of the current study has an elliptical cross-sectional apical shape and dimension within the range of existing root canal models. Compared to the existing models, the canal length is shorter and has a value of 10.2 mm. However, the working length refers only to the actual canal section, so this length is also comparable with lengths of existing models.

The main advantage of the demonstrated approach for the determination of an artificial root canal model based on the statistically evaluated root canal’s length, curvature, torsion and cross-sectional dimensions as introduced by Kucher [23] is that the obtained model includes the entirety of the root canals examined. This would improve the representational capability of the artificial root canal models. Currently, this approach is only applied to a particular kind of root canal without any ramification. However, the investigation seems to be adaptable to other kinds of root canals. Furthermore, an analysis of root canals with ramifications is possible. However, a mathematical description considering the individual sections between the ramifications has to be developed. Therefore, the classification method described by Ahmed et al. [73] could be a good basis. Nevertheless, the demonstrated approach provides a valuable method for generating more realistic artificial root canal models that allow more realistic endodontic investigation and results in better training teeth and blocks for endodontic teaching. Therefore, new developments in the field of additive manufacturing enable the fabrication of these advanced 3D root canal models. Furthermore, the obtained geometry can be used to design CAD models for the fabrication endodontic training blocks made of bovine dentine with more realistic mechanical properties (compare [6]). In this way, optimal experimental conditions for endodontic research and equitable student education that is as natural as possible are reached.

## 5. Conclusions

In endodontics, there several applications which require an artificial root canal model. Using the statistical evaluation of the geometrical properties of length, curvature, torsion and cross-sections of unbranched root canals, a geometry-based root canal model can be determined. On the basis of this approach, the volume models of teeth were obtained by computer tomographic scans in the microscopic scale. By reconstructing an average center line, the average cross-sectional dimensions and the average root canal’s torsion, a 3D model can be generated which represents the entirety of the root canals examined. In this way, the presented approach will enable the creation of improved artificial root canal models and a 3D printable geometry for endodontic investigations and pre-clinical endodontic training use in the future.

## Figures and Tables

**Table 2:** Evaluation of the center line’s radius of curvature (considered for lower confidence interval and the whole canal length).

Property	Unit	Value
Mean value	mm	6.1
Minimum	mm	2.5
Maximum	mm	13.4

## Data Availability

Not applicable.

## Supplementary Materials

The following supporting information can be downloaded at: https://www.mdpi.com/article/10.3390/dj11050133/s1, CAD data: 3D-printable training block.

## Appendix A

Using the 3D geometry of the resulting artificial root canal model according to Section 3.2, an endodontic training block with the dimensions of 8.9 mm × 5 mm × 5 mm was realized (Figure A1). The CAD model of this training block is provided as supplementary material.
